# Proteomic study of evolved *Pseudomonas aeruginosa* strains grown in *Staphylococcus aureus*- and *Klebsiella pneumoniae*-conditioned media

**DOI:** 10.1128/msystems.00111-25

**Published:** 2025-06-03

**Authors:** Yanrong Pan, Tin Yan Wong, Jordy Evan Sulaiman, Henry Lam

**Affiliations:** 1 Department of Chemical and Biological Engineering, The Hong Kong University of Science & Technology, Kowloon, Hong Kong; 2 Department of Health Technology and Informatics, The Hong Kong Polytechnic University 26680, Hong Kong, Hong Kong; University of California Irvine, Irvine, California, USA

**Keywords:** *P. aeruginosa*, *S. aureus*, *K. pneumoniae*, supernatant, laboratory evolution, proteomics

## Abstract

*Pseudomonas aeruginosa* (PA) is an opportunistic pathogen that causes multiple infections. Co-infections of PA with *Staphylococcus aureus* (SA) and *Klebsiella pneumoniae* (KP) were frequently reported in severe community-acquired pneumonia. However, it is challenging to mimic such natural conditions and maintain a long-term stable community of these pathogens in the laboratory, thus limiting the study of PA’s adaptation in the presence of SA and KP. In this study, we performed adaptive laboratory evolution (ALE) using a simplified supernatant-based culture model to explore the evolution of PA under the biochemical influences of SA and KP and elucidate the adaptation mechanism of the evolved PA strains using proteomics. After 15 growth cycles with the cell-free supernatants of SA (SASn) and KP (KPSn), PA displayed significant alterations in phenotypes, including motility, antibiotic sensitivity, toxicity, biofilm formation, pyocyanin production, relative fitness, and growth profile. Whole-genome sequencing revealed nonsynonymous mutations in the SASn- and KPSn-evolved PA strains in *ampG*, *dipA*, *anmK*, *bifA*, and *rpoS* genes, which were not observed in the evolved strains that were cultured in the absence of SA/KP supernatants. Notably, the supernatant-evolved mutants exhibited differential regulation of key pathways (type VI secretion system, biofilm formation, phenazine biosynthesis, translation, β-lactam resistance, and O-antigen biosynthesis) compared to the ancestral strain and the unmodified medium-evolved (UmMd-evolved) strain. Our results suggested that adaptive evolution in such supernatant-based pseudo-coculture models can be a viable strategy to gain insights into how PA adapts under the influence of other pathogens, which may have clinical implications in understanding and controlling co-infections.

**IMPORTANCE:**

Through the supernatant-based ALE approach, we examined the evolutionary adaptations of PA upon repetitive growth cycles in cell-free supernatants of SA and KP. Compared to the unmodified medium-evolved (UmMd-evolved) strain, the SA- and KP supernatant-evolved (SASn- and KPSn-evolved) strains acquired distinct mutations and exhibited different phenotypic and proteomic alterations. The SASn- and KPSn-evolved PA strains display elevated cytotoxicity and enhanced competitiveness against SA and KP compared to the ancestral strain. SASn- and KPSn-evolved PA strains displayed some similarities in terms of the proteomic profile, especially in the expression of type VI secretion system (T6SS). Both SASn- and KPSn-evolved PA strains positively and negatively regulated H2 and H3-Hcp secretion islands (HSIs) of T6SS, respectively, while the UmMd-evolved strain negatively regulated both H2 and H3-T6SS. These suggest the potential role of SA and KP in modulating the regulation of T6SS HSIs in PA.

## INTRODUCTION

*Pseudomonas aeruginosa* (PA) is an opportunistic and multidrug-resistant pathogen that causes many clinical infections (1). PA frequently coexists with *Staphylococcus aureus* (SA) (2) and *Klebsiella pneumoniae* (KP) (3) in clinical polymicrobial infection cases, such as cystic fibrosis (CF), pneumonia, urinary tract infections, and wound infections (4–9). These cases are associated with poor clinical outcomes and increased antibiotic resistance and tolerance (9, 10). With synergistic interactions among multiple pathogens, the duration of infections tends to lengthen and worsen, underscoring the importance of investigating prevalent combinations of bacterial infections (11).

Extensive research has been carried out on the interaction between PA and SA, revealing certain molecular mechanisms. For instance, *Staphylococcal* protein A (SpA) interacts with PA, facilitating aggregation through Psl and type IV pili binding (12), while PA hinders SA electron transport using 2-n-heptyl-4-hydroxyquinoline N-oxide (HQNO) and siderophores (13). In addition, physical interactions between cells of different species, such as those facilitated by contact-dependent systems like the T6SS, as well as chemical interactions, such as toxin interference, have been described in communities involving PA, SA, and KP (14–16). Although PA is known to be an antagonistic partner in planktonic co-cultures, it also engages in cooperative behavior in biofilms by secreting extracellular polymeric substances (EPS) and siderophores that benefit neighboring cells (17, 18). A potential synergistic relationship between PA and SA was reported, involving metabolic modulations that affect pathogen colonization, virulence, as well as antibiotic resistance and tolerance (18–20). In rich media, PA can coexist with KP, but when facing iron limitation, it displaces KP by employing rhamnolipid biosurfactant (21).

Many *in vitro* studies only focused on short-term PA co-culture with other species (2, 22–24), partly because in planktonic cultures, PA rapidly outcompetes its neighboring species (e.g., SA [23] and KP [22]) and becomes the dominant species in the community. Thus, laboratory co-cultures involving PA are unstable and difficult to control. Yet, it is known that they form relatively stable communities in clinical infection cases with SA and KP (8), though PA may gradually outcompete and establish dominance over SA and KP in some cases (25, 26). Therefore, a simple laboratory co-culture model that allows long-term investigation of the adaptation mechanisms of PA in the presence of other species is needed.

Previous studies co-cultured PA with either SA (24, 27) or KP (28) within biofilms to obtain relatively stable dual-bacterial biofilm models. For example, Muzaki et al. demonstrated that deleting *lsrB*/*lsrD* (autoinducer-2 transporter genes) in KP altered its interaction with PA from cooperative to competitive, and PA monospecies biofilm formation can be promoted when cultured in the supernatant of the ∆*lsrB*/∆*lsrD* KP mutant (29). Through short-term biofilm co-culture (18 h) and proteomic analysis, Reigada et al. found that PA’s motility was increased in the presence of SA, but the abundance of pigments of PA (pyocyanin and pyoverdine) was decreased (24). To solve the stability issue and to observe the longer-term behavior of PA under the influence of other species in planktonic cultures, Tognon et al. employed a method of continuously introducing fresh SA to growing PA culture for 15 days, focusing on the phenotypic and genomic changes of PA under the presence of SA (30). The evolved PA strain obtained mutations leading to lipopolysaccharide (LPS) deficiency and displayed increased resistance to β-lactams (30). In another study, Niggli et al. conducted similar evolution experiments on SA by growing it in the supernatant of PA, thus focusing on how PA influences the long-term growth of SA (31). They found that the *Pseudomonas* quinolone signal (PQS) pathway was involved in the inhibition of SA growth, and SA eventually became resistant to the inhibition by PA’s supernatant after prolonged treatment cycles. This “community module” approach, which grows bacteria with the fresh supernatants of other species in the long term, could solve the problem of unstable co-cultures involving PA and reveal the molecular mechanism of PA’s adaptation in communities (23, 24, 32, 33).

Previous studies used whole-genome sequencing (WGS) to identify mutations in the evolved strains. However, solely utilizing WGS without follow-up systematic analyses to elucidate the mechanisms underlying the evolutionary adaptations may not capture indirect biological alterations due to the mutations. To get the full picture of the adaptation mechanisms of the evolved strain after such evolution experiments, transcriptomic and/or proteomic profiling could be employed. Transcriptomic profiling by RNA-seq can be applied to study bacterial adaptation mechanisms (34), but mRNAs have shorter lifespans in bacteria, and not all mRNAs will be translated into proteins (35–37). By contrast, proteomics directly measures proteins, which are chiefly responsible for the observed phenotype at a given time. Proteomics has been shown to successfully generate insights into the adaptation mechanisms of laboratory-evolved strains of various species (e.g., *E. coli* and SA) under diverse experimental conditions (38–44).

In this study, we performed adaptive laboratory evolution (ALE) using bacterial supernatants of SA and KP (SASn and KPSn, respectively) to explore the evolutionary adaptations of PA under the influence of the two pathogens. This approach generated two mutant strains that differed significantly in phenotypes from the ancestral strain and strain cultured in the absence of SA/KP supernatants (unmodified medium-evolved strain or UmMd-evolved strain). The SASn- and KPSn-evolved strains exhibited increased biofilm formation, enhanced pyocyanin production, and reduced motility. By subjecting the evolved strains to proteomic analysis, we revealed variations in the proteome of the SASn- and KPSn-evolved strains compared to the UmMd-evolved strain. Further, although the mutations in the SASn- and KPSn-evolved strains are not directly related to virulence, they resulted in increased cytotoxicity and enhanced growth competitiveness with SA and KP. The SASn-evolved PA strain also exhibited increased ampicillin sensitivity. In summary, our supernatant-ALE approach combined with proteomics elucidated important phenotypes and pathways associated with PA’s evolutionary adaptation under the influence of other pathogens and revealed key mutations that are potentially associated with increased virulence.

## RESULTS

### SASn- and KPSn-evolved strains displayed increased biofilm formation, increased pyocyanin production, and reduced motility

**Fig 1:**
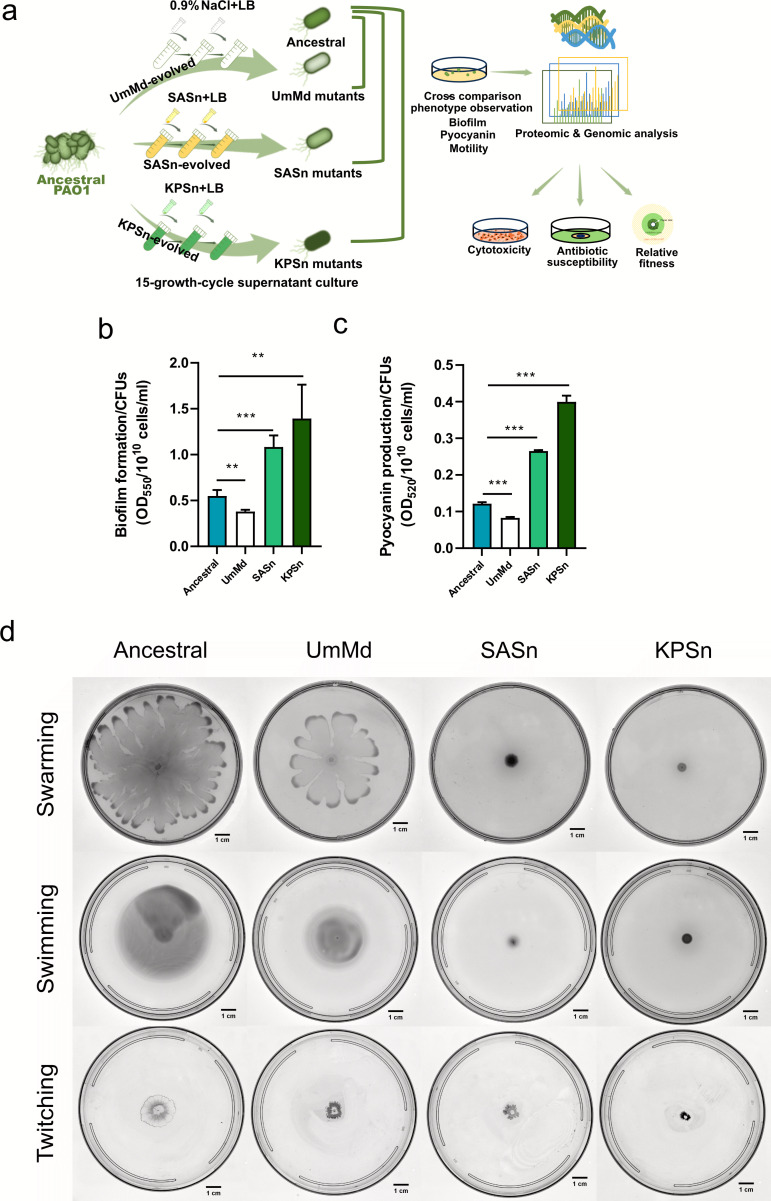
Phenotypic changes of the evolved strains of PA. Phenotypic changes in PA after (a) 15-cycle serial passaging in the LB medium supplemented with fresh NaCl solution (UmMd-evolved), SA supernatant (SASn-evolved), and KP supernatant (KPSn-evolved). (b) Biofilm formation of the evolved strains and ancestral strain, normalized by bacterial colony-forming units (CFUs) (mean ± s.d., *n* = 4). (c) Pyocyanin production of the evolved strains and ancestral strain (mean ± s.d., *n* = 4), normalized by bacterial CFUs. (d) Motility of the evolved strains and ancestral strain (swarming, swimming, and twitching). Significance of difference with the ancestral strain: ns, not significant, ∗*P* < 0.05, ∗∗*P* < 0.01, and ∗∗∗*P* < 0.001 (two-tailed *t*-test with unequal variances). The scale bar is 1 cm.

PA was cultured in Luria-Bertani (LB) broth medium supplemented with 30% supernatant from either SA (SASn) or KP (KPSn) for 15 growth cycles (48 h per cycle) (31). As a control, the UmMd-evolved group was treated with 0.9% sodium chloride (NaCl) solution. The experimental setup and timeline of the evolution study are depicted in Fig. 1a. The evolved PA populations (SASn-, KPSn-, and UmMd-evolved) showed distinct phenotypes compared to the ancestral strain. Single colonies from these evolved populations were further analyzed. SASn- and KPSn-evolved strains exhibited increased biofilm formation compared to the ancestral strain, whereas the UmMd-evolved strain shows decreased biofilm formation (Fig. 1b). Pyocyanin production was slightly decreased in the UmMd-evolved strain group, but significantly higher in both SASn- and KPSn-evolved groups (Fig. 1c). Motility capabilities (swarming, swimming, and twitching) were lower in all three evolved strains than the ancestral PA, but the decrease in motility was more significant in the SASn- and KPSn-evolved strains (Fig. 1d). The three evolved strains were further cultured in the LB medium over 14 days (seven growth cycles) using a protocol analogous to the supernatant-based evolution. There was no significant change in biofilm formation or pyocyanin production, indicating that the phenotypic changes in the evolved strains were persistent. (Fig. S1).

### Whole-genome sequencing revealed nonsynonymous mutations in the evolved strains

To reveal the genetic changes in the evolved PA mutants, after 15 growth cycles, we subjected both the evolved mutants and the populations where the mutants were isolated to WGS. Single-nucleotide polymorphisms (SNPs) and insertions/deletions (indels) relative to the ancestral strain were identified. Only six nonsynonymous mutations were found at above 40% frequency in the population and detected in the isolated SASn- and KPSn-evolved strains (Table 1; Fig. S2). Two more nonsynonymous mutations were detected in the KPSn-evolved population at above 15% variant allele frequency (VAF), but were not found in the isolated strain (Table S1). By comparison, the UmMd-evolved strain has accumulated 16 nonsynonymous mutations in various genes, and another 14 nonsynonymous mutations were detected in the population at above 15% VAF but were not found in the isolated strain (Table S2).

**TABLE 1:** Nonsynonymous mutations identified in Sn-evolved strains of PA^*a*^

Strain	Position	REF	ALT	Protein change	Gene	VAF (%) of population	VAF (%) of isolated strains
KPSn	718402	G	A	Glutamine (Q) → stop Loss of 233 amino acids	*anmK*	61.2	100
	4058007	TC	T	Loss of 20 amino acids*	*rpoS*	67.8	100
	4896210	TTCCTCGCTGAGC	T	Deletion of four amino acids	*bifA*	47.3	100
SASn	4058609	C	A	Serine (S) → isoleucine (I)	*rpoS*	98.8	100
	4923646	GTCAGCAAGGTCTTCGGCGTACTCATGACCCT	G	Loss of 172 amino acids*	*ampG*	50.0	100
	5643032	GA	G	Loss of 477 amino acids*	*dipA*	96.2	100

^*a*^ Asterisks indicate nucleotide deletion that results in a frameshift and a premature stop codon downstream of the deletion.

As expected, the VAF values of the detected mutations in the isolated strains were 100%, whereas the VAF values for mutations detected in the populations varied, indicating that the populations were still heterogeneous at the end of the evolution experiment. Nonetheless, given the high VAF values of the six nonsynonymous mutations in population, the isolated strains were the representative strains in the respective evolved populations. Hereafter, we limit our attention to only the nonsynonymous mutations found in the supernatant-evolved strains.

The mutated genes in the SASn- and KPSn-evolved strains were all different. Interestingly, both the SASn- and KPSn-evolved strains acquired mutations in different regions of the same gene, *rpoS*. RpoS functions as a common regulator for the general stress response and acts as a transcription factor for genes involved in various processes including motility, biofilm formation, antibiotic tolerance, and virulence (45). These mutations suggested that the stress regulation of PA was affected during growth in the supernatant of bacterial competitors. In the KPSn-evolved strain, the mutation in *rpoS* (single-base deletion) led to the appearance of a premature stop codon in the middle of the gene. By contrast, the SNP in the SASn-evolved strain resulted in the substitution of an amino acid from serine to isoleucine at this site (Table 1; Fig. S2).

In the SASn-evolved strain, we also observed a single-base deletion in the *ampG* gene. AmpG is a transmembrane protein responsible for transporting signal molecules that induce the expression of the β-lactamase AmpC (46). Inactivation of AmpG drastically represses the intrinsic β-lactam resistance. The loss of this segment of bases results in premature termination of *ampG*, which may lead to increased sensitivity toward ampicillin. Another base deletion occurred in *dipA*, a phosphodiesterase that plays an essential role in biofilm dispersal (47), leading to the premature appearance of a stop codon. The inactivation of DipA has been reported to cause a deficiency in biofilm dispersion, affect the architecture of PA biofilms and c-di-GMP levels, impair swarming and swimming motility, but enhance initial attachment and Psl polysaccharide production for biofilm formation (47). Therefore, the increased biofilm formation and decreased motility observed in the SASn-evolved strain may be due to the mutation in *dipA* (Fig. 1b and d).

In the KPSn-evolved strain, BifA, which is a membrane protein that exhibits phosphodiesterase activity but no detectable diguanylate cyclase activity, is mutated. Inactivation of BifA was shown to enhance biofilm formation and decrease flagella reversals (48), which are involved in swarming. Since the mutation in *bifA* is a single-base deletion that introduced the termination codon early in the gene, the decreased swarming ability of the KPSn-evolved strain might be related to the *bifA* mutation. Another mutation occurred in the *anmK* gene, which plays a role in the pathogen’s peptidoglycan metabolism (49). The inactivation of AnmK leads to impaired growth (49), increased β-lactam susceptibility (49), decreased biofilm formation, and loss of twitching motility (50). Here, the single-point mutation on *anmK* resulted in the premature termination of the gene due to amino acid substitution to a stop codon. This may potentially inactivate AnmK function and lead to the aforementioned changes in the phenotype.

In summary, diverse mutations were found in the evolved strains, each with plausible connections to the observed changes in their phenotypes, such as antibiotic resistance, biofilm formation, and motility. It is important to note that adaptive evolution is based on random mutagenesis followed by selection, and thus there may be multiple evolutionary pathways for PA to adapt to the influence of SA and KP.

### Global analysis of protein regulation in the evolved PA strains

To uncover the underlying adaptation mechanisms of the UmMd-evolved, SASn-evolved, and KPSn-evolved PA strains, we conducted label-free proteomics on the ancestral strain and evolved strains, cultured under their respective conditions as in the evolution experiment. A total of 3,890 unique proteins were detected across all sample groups. To assess the similarities in the proteome profile between sample groups, we performed principal component analysis (PCA) on the protein abundances, as estimated by the normalized spectral abundance factor (NSAF). The proteome profiles of the UmMd-evolved strain, ancestral strain, and two Sn-evolved strains are positioned uniquely, while the protein profiles of the two Sn-evolved strains are closely positioned (Fig. S3).

**Fig 2:**
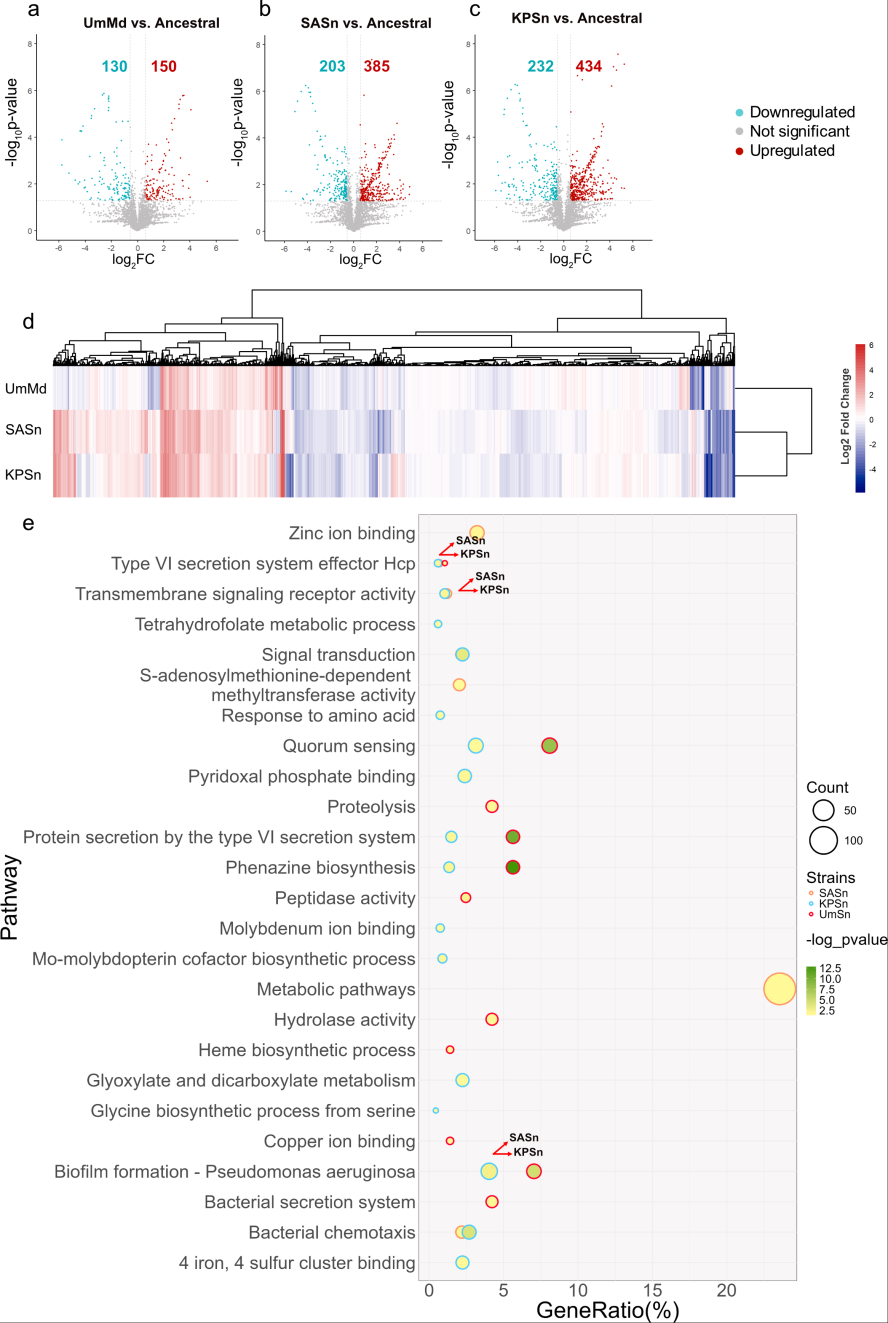
Proteome profile of the SASn-evolved strain, KPSn-evolved strain, UmMd-evolved strain, and ancestral strain. (**a–c**) Volcano plots of UmMd-evolved, SASn-evolved, and KPSn-evolved strains compared to the ancestral strain. The ancestral strain was cultivated in LB broth, while the other three strains were cultured in the respective evolution experiment media. Differentially expressed proteins (DEPs) are identified as proteins with *P*-values less than 0.05 and absolute fold changes exceeding 1.5, delineating specific regions. The left regions denote proteins exhibiting downregulation, while the right regions represent upregulated proteins. (d) Heatmap of the DEPs across the UmMd-evolved strain, SASn-evolved, and KPSn-evolved strains. Hierarchical clustering was performed using Euclidean distance and a ward linkage model. (e) Bubble chart for GO, InterPro, KEGG, pathway enrichment analysis, and classified by DAVID; *y*-axis: pathway names, *x*-axis: gene ratio, bubble size: gene count, bubble color: -log *P*-value of pathways, and border color of the bubbles: strains.

We define differentially expressed proteins (DEPs) as those with fold change higher or lower than 1.5 and −1.5 relative to the ancestral strain and *P*-value less than 0.05. In the SASn-evolved strain, 385 proteins were upregulated, and 203 proteins were downregulated compared to the ancestral strain (Fig. 2a). Similarly, there were 434 upregulated proteins and 232 downregulated proteins in the KPSn-evolved strain compared to the ancestral strain (Fig. 2b). On the other hand, the number of differentially expressed proteins is lower in the UmMd-evolved strain than in the ancestral strain (150 proteins were upregulated and 130 proteins were downregulated) (Fig. 2c). Based on the heatmap of fold changes of the DEPs in the Sn-evolved strains and UmMd-evolved strain (Fig. 2d), we observed that the DEPs in the SASn-evolved strain and KPSn-evolved strain are clustered together, while the DEPs in the UmMd-evolved strain differed from the other groups. The Venn diagram of the DEPs among the three evolved strains (Fig. S4) also shows that there are more common DEPs between the SASn- and KPSn-evolved strains than there are between either Sn-evolved strains and the UmMd-evolved strain.

Functional annotation and pathway enrichment analysis of all differentially expressed proteins (DEPs) led to the identification of common pathways and functions among the three evolved strains, as depicted in Fig. 2e. All three evolved strains exhibited regulations in the T6SS effector Hcp and biofilm formation. Additionally, both Sn-evolved strains demonstrated regulations in transmembrane signaling receptor activity, signal transduction, and bacterial chemotaxis. UmMd- and KPSn-evolved strains displayed regulations in phenazine biosynthesis, protein secretion by the T6SS, and quorum sensing. Notably, in the SASn-evolved strain, DEPs related to metabolic pathways exhibited a high ratio and count. The analysis of these DEPs in conjunction with the phenotypes and genetic mutations of evolved strains revealed that the mutated genes not only altered biofilm formation, pyocyanin production, and motility but may also influence bacterial competitiveness (via changes in T6SS-related proteins), growth (via metabolism-related protein changes), and interspecies interactions (via quorum sensing-related protein variations), mediated by biochemical cues in the secretomes of SA and KP.

### The proteomic profile comparison between different evolved strains

**Fig 3:**
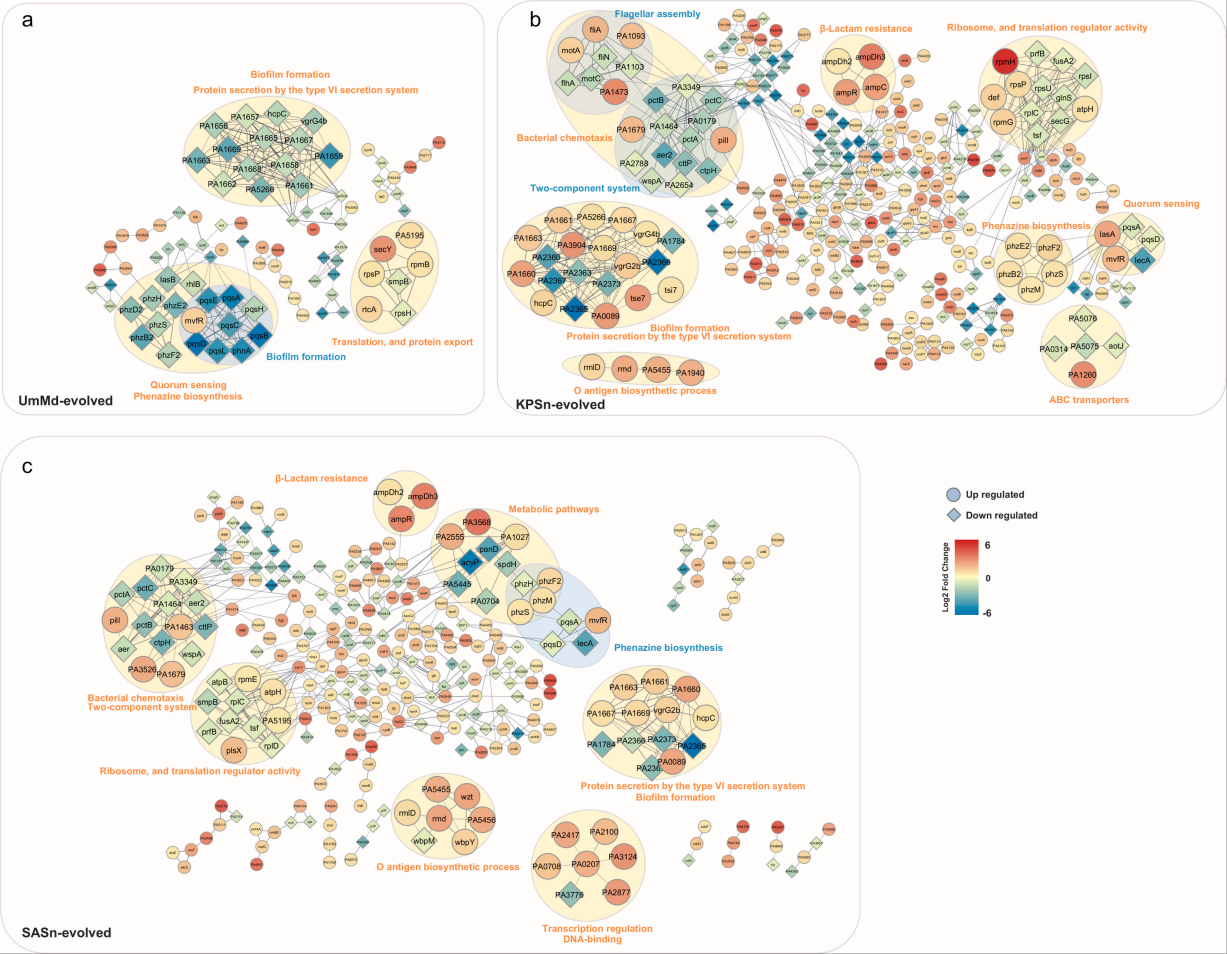
Global analysis of protein regulation in evolved bacterial strains. (**a–c**) Protein–protein interaction (PPI) network of the UmMd-evolved strain, KPSn-evolved strain, and SASn-evolved strain compared to the ancestral strain, analyzed by STRING. Circular nodes indicate upregulation of protein expression compared to the ancestral strain, while diamond nodes represent downregulation. The colors show the values of log_2_ fold change. The lines represent protein interaction (thicker lines mean higher confidence). All proteins in the figure were identified with *P*-values less than 0.05 and absolute fold changes exceeding 1.5.

DEPs were further analyzed through the protein-protein interaction (PPI) networks, clustering based on pathways and functions with the upregulation and downregulation of individual proteins and their fold changes (Fig. 3a through c).

Compared to the ancestral strain, the UmMd-evolved strain demonstrated decreased protein expression (Fig. 3a) related to biofilm formation, T6SS-mediated protein secretion, phenazine biosynthesis, and quorum sensing (except for MvfR). These regulations might be connected to the decreases in biofilm and pyocyanin production in the UmMd-evolved strain (Fig. 1). Conversely, many proteins involved in translation and protein export showed increased expression levels (except for RpsH and SmpB).

In the KPSn-evolved strain, DEPs related to phenazine biosynthesis, O-antigen biosynthesis, and β-lactam resistance exhibited upregulations (Fig. 3b). The upregulation of DEPs in phenazine biosynthesis may be associated with the increased production of pyocyanin in the KPSn-evolved strain.

In the SASn-evolved strain, DEPs related to β-lactam resistance exhibited upregulation, along with all DEPs; except PA3776, DEPs in DNA binding and transcription regulation are upregulated (Fig. 3c). Furthermore, DEPs involved in O-antigen biosynthesis were upregulated, with the exception of WbpM (Fig. 3c). The downregulation of WbpM linked to B-band synthesis (51, 52), and upregulation of proteins (Rmd, Wzt, and WbpY) associated with A-band synthesis (52) was observed only in SASn-evolved strains. The LPS of PA comprises two types of O-antigens known as A and B bands (53). The variant regulations of the A-band, highly conserved in PA serotypes, and the B-band, varying in composition among different serotypes, may impact the pathogenicity, virulence, and host immune response of PA through their differential expression (54).

**Fig 4:**
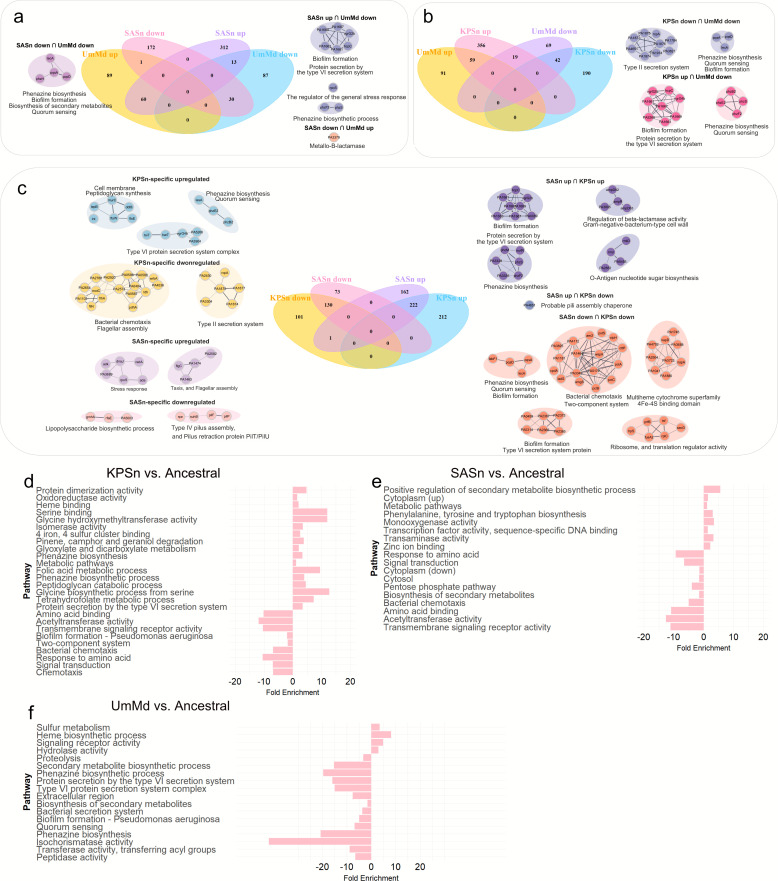
The proteome profile comparison between different evolved strains. (**a–c**) Analysis of overlapping and strain-specific proteins. (**d–f**) Enrichment analysis of upregulated and downregulated proteins in each evolved strain, with their fold enrichment displayed on the *x*-axis. Negative values indicate downregulation, while positive values indicate upregulation. All proteins were identified with *P*-values less than 0.05 and absolute fold changes exceeding 1.5.

By focusing on the overlapping DEPs and the unique DEPs in each evolved strain, we identified certain groups of proteins that exhibit specific regulations within the same pathways or functions across different bacterial strains (Fig. 4a through c).

The proteins associated with biofilm formation and protein secretion by the T6SS (including HcpC, PA1669, VgrG2b, PA1661, PA1667, and PA1663) exhibited upregulation in the SASn- and KPSn-evolved strains but downregulation in the UmMd-evolved strain (Fig. 4a and b). The expression of *rpoS*, responsible for stress response, was upregulated in the SASn-evolved strain but downregulated in the UmMd-evolved strain. The expression of proteins involved in the type II secretion system, which is related to cytotoxicity and adherence to host cell surfaces (55), decreased in both the KPSn-evolved strains and UmMd-evolved strain, but there was no significant change in the SASn-evolved strain (Fig. 4c). There was a unique upregulation of proteins related to peptidoglycan synthesis and cell membrane proteins (including Lnt, LepB, DdlB, MurC, TsW, and FtsE) in KPSn-evolved strains.

The DEPs observed on the Hcp secretion island (HSI) in the evolved strains are noteworthy as HSI is linked to the virulence of PA. HSI consists of three gene clusters, H1-, H2-, and H3-T6SS, encoding the components of the T6SS (56). These clusters are believed to have distinct functionalities and can be regulated through different mechanisms (1). However, there is currently limited research on the mechanisms of H2- and H3-T6SS. Interestingly, while both H2- and H3-T6SS were downregulated in the UmMd-evolved strain, in both SASn- and KPSn-evolved strains, the proteins belonging to H2-T6SS showed upregulation (SASn- and KPSn-evolved strains: HsiG2, HsiH2, Sfa2, HsiJ2, IcmF2, and VgrG2b), while the proteins belonging to H3-T6SS were downregulated (SASn-evolved strain: HsiB3, HsiC3, Hcp3, and VgrG3; KPSn-evolved strain: HsiA3, HsiJ3, HsiB3, HsiC3, Hcp3, and VgrG3) (1, 57). The MvfR protein was found to be upregulated in all three evolved strains. The MvfR protein, which typically has a negative effect on H1-T6SS transcription and a positive effect on the transcription of H2- and H3-T6SS (57), did not follow the expected pattern based on the differential regulation of the T6SS systems between the SASn- and KPSn-evolved strains and the UmMd-evolved strain. Thus, the observed upregulation of MvfR protein in all three evolved strains could indicate alternative regulatory mechanisms that override the typical MvfR-mediated transcriptional effects on the T6SS clusters (1, 57)

Additionally, the protein AmpDh3, which is delivered by H2-T6SS, was also found to be upregulated in both SASn- and KPSn-evolved strains. AmpDh3 plays a crucial role in hydrolyzing peptidoglycans located on the cell wall of the prey bacterium, leading to prey cell death, and it provides an evolutionary advantage for PA in the competition (1).

By comparing the fold enrichment of the evolved strains (Fig. 4d and e), we observed that metabolic pathways were mainly upregulated in KPSn-evolved strains and significantly downregulated in the UmMd-evolved strain. Pathways related to amino acids, such as response to amino acid and amino acid binding, showed significant downregulation in the SASn- and KPSn-evolved strains. On the other hand, tetrahydrofolate metabolic process and glyoxylate and dicarboxylate metabolism were significantly upregulated in the KPSn-evolved strain. Both of these pathways are related to energy production and carbon source utilization, and they are unique to the KPSn-evolved strain. Besides, proteins related to the T6SS are significantly downregulated in the UmMd-evolved strain, while they are significantly upregulated in the KPSn-evolved strain. In competition with other bacteria, the T6SS in PA serves as an efficient antibacterial weapon (1), suggesting that the KPSn-evolved strain may have a competitive advantage over other bacteria in co-culture.

### SASn- and KPSn-evolved PA strains exhibited altered growth profiles and higher relative fitness compared to the ancestral and UmMd-evolved strains

**Fig 5:**
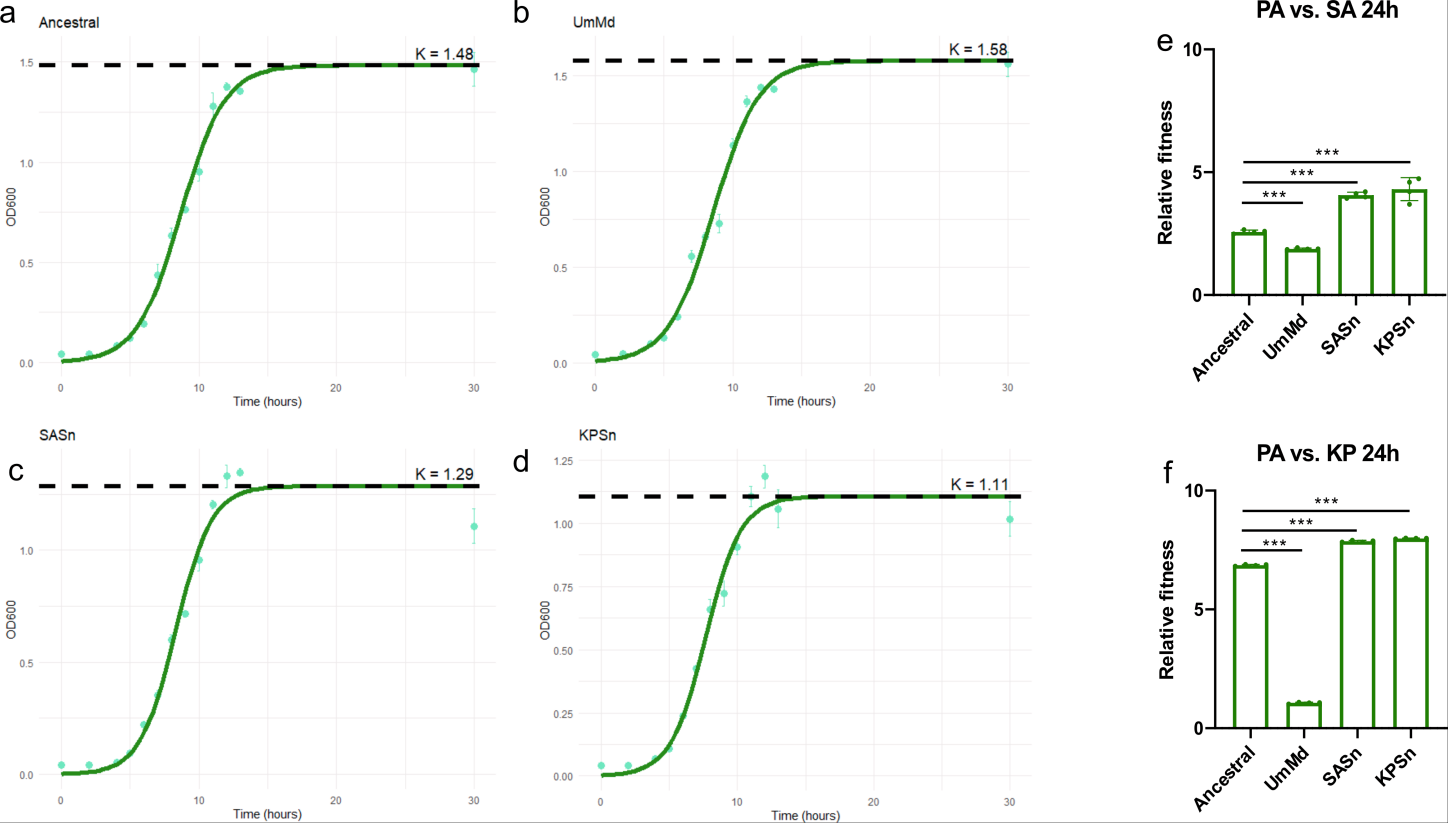
The growth curve and relative fitness of evolved PA strains and ancestral strain. (**a–d**) 30 h growth curve of the four strains (mean ± s.d., *n* = 10), fitted to the logistic growth model to estimate (green curves) the maximum carrying capacity, K. (**e, f**) Relative fitness of the evolved PA strains and ancestral strain, cocultured with SA and KP for 24 h (mean ± s.d., *n* = 4). Significance of difference with the ancestral strain: ns, not significant, ∗*P* < 0.05, ∗∗*P* < 0.01, and ∗∗∗*P* < 0.001 (two-tailed *t*-test with unequal variances).

From our proteomics analysis, we detected DEPs related to transcription, cell membrane, and DNA replication in the evolved strains. Thus, we speculate that the growth profile of the evolved strains will be altered in comparison to the ancestral strain. We monitored the growth of the ancestral strain and the UmMd-, SASn-, and KPSn-evolved strains by measuring OD_600_ at 0, 2, 4, 5, 6, 7, 8, 9, 10, 11, 12, 13, and 30 h to capture all growth phases. Growth curves were fitted for each strain, with the maximum carrying capacity (K), defined as the maximum population density achievable under experimental conditions, serving as a key quantitative parameter. The results revealed distinct evolutionary outcomes (Fig. 5a through d); while the SASn- and KPSn-evolved strains failed to reach the ancestral strain’s K, the SASn-evolved strain exhibited closer proximity to ancestral strain levels. In contrast, the UmMd-evolved strain surpassed the ancestral strain with a slightly higher K value. We co-cultured the ancestral, UmMd-evolved, SASn-evolved, and KPSn-evolved PA strains with SA or KP for 24 h to observe differences in their competitive ability. Despite having the highest maximum carrying capacity among the evolved strains, the UmMd-evolved strain exhibited the lowest relative fitness in both liquid and solid media (Fig. 5b and c; Fig. S5a through d). On the other hand, both the SASn-evolved and KPSn-evolved strains demonstrated higher relative fitness compared to the ancestral strain, despite their lower carrying capacity.

### The SASn and KPSn-evolved PA strains displayed increased cytotoxicity and decreased ampicillin sensitivity

**Fig 6:**
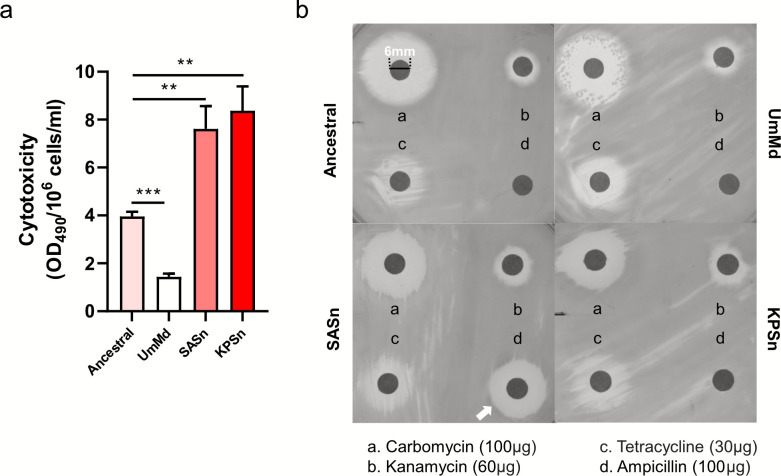
Cytotoxicity and antibiotic susceptibility of evolved strains and the ancestral strain. (a) Cytotoxicity of HeLa cells infected with PA after 10 h (mean ± s.d., *n* = 3), measured through the LDH level of cells, normalized by colony-forming units (CFUs) within the wells at the 10 h time point of ancestral and evolved PA. Significance of difference: ns, not significant, ∗*P* < 0.05, ∗∗*P* < 0.01, and ∗∗∗*P* < 0.001 (two-tailed *t*-test with unequal variances). (b) Antibiotic susceptibility of the four strains.

Our proteomics data showed that the SASn- and KPSn-evolved strains exhibited substantial changes in the expression of proteins associated with toxicity and invasiveness (Fig. 3 and 4). We co-cultured HeLa cells with SASn-, KPSn-, UmMd-evolved strain, and ancestral strain for 24 h at the multiplicity of infection (MOI) of 100 in every group. The lactate dehydrogenase (LDH), which can be released into the supernatant upon cell membrane damage, was used as a marker to measure the cytotoxicity of PA strains (see Materials and Methods for details). Of all strains, the UmMd-evolved strain demonstrated the lowest cellular toxicity (Fig. 6a). By contrast, the SASn-evolved and KPSn-evolved strains exhibited significantly higher cellular toxicity compared to the ancestral strain. Our results demonstrate that evolutionary adaptation of PA toward the influence of another species could indirectly impact its virulence and its interaction with the host.

In addition to proteins related to cytotoxicity, our proteomic analysis also showed the upregulation of β-lactam resistance-associated proteins. Besides, the SASn- and KPSn-evolved strains also acquired mutations in antibiotic resistance-related genes (*anmK* and *ampG*). To see whether the evolved strains displayed altered sensitivity to antibiotics, we conducted antibiotic susceptibility testing using the disk diffusion assay (Fig. 6b; Fig. S6). Of all evolved strains, only the SASn-evolved strain exhibited an increase in sensitivity to ampicillin (the average of the zone diameters of the four replicates is 15.98 mm compared to 0 mm for other strains). This increase in susceptibility to ampicillin in the SASn-evolved strain might be due to the mutation in the *ampG* gene.

## DISCUSSION

Multiple bacterial infections involving PA are often fatal (58). Additionally, SA and KP, as two of the top five major bacterial pathogens, frequently coexist in PA infection cases (8, 58). Therefore, studying their interactions is essential. However, long-term coculture *in vitro* is challenging due to the tendency of PA to outcompete SA and KP (2, 22–24). In this study, we employed laboratory evolution using fresh bacterial supernatants to investigate the growth dynamics and evolutionary adaptation of PA in response to the secretomes of SA and KP. Using this approach, we obtained evolved PA strains from the respective supernatants. The WGS and proteomic investigation of the supernatant-evolved PA mutants elucidates the alteration in protein regulations and interactions due to the mutations in the genome.

Compared to the ancestral and UmMd-evolved strains, both SASn- and KPSn-evolved strains exhibit increased biofilm formation (Fig. 1b), pyocyanin production (Fig. 1c), relative fitness (Fig. 5b and c), and cytotoxicity (Fig. 6a), and they display decreased motility (Fig. 1d). The SASn-evolved strain uniquely exhibits increased β-lactam sensitivity (Fig. 6b; Fig. S6), and the KPSn-evolved strain acquired decreased maximum carrying capacity (Fig. 5a). These phenotypes are associated with mutations in PA. For instance, the mutation in *anmK* may result in loss of twitching motility and impaired growth of KPSn-evolved strains. Although the inactivation of AnmK may also cause increased β-lactam susceptibility and reduced biofilm formation (49), the presence of other mutations, like *rpoS*, may lead to epistasis, thus amplifying the impact on these pathways. Besides, the deletion of bases on the *bifA* gene could lead to its inactivation, thereby increasing biofilm formation. Consequently, biofilm formation in the KPSn-evolved strain shows an increase due to epistasis, while β-lactam susceptibility remains unchanged. In the SASn-evolved strain, mutations in the *dipA* gene may result in the lack of biofilm dispersion, leading to an increase in the total biomass of the biofilm. Upregulation of β-lactam resistance-related proteins was observed in both SASn- and KPSn-evolved strains. However, only the SASn-evolved strain showed increased sensitivity to ampicillin (Fig. 6b; Fig. S6), which is linked to the loss of 31 base pairs in the *ampG* gene (which resulted in premature termination of transcription), potentially leading to gene inactivation. This mutation and protein regulation might be a way for PA to maintain metabolism and fitness costs in the absence of antibiotics (59).

The mutations in the UmMd-evolved strain do not overlap with those found in the SASn- and KPSn-evolved strains. Functional analysis revealed that mutated genes are related to metabolism, signaling, membrane components, and other functions (Table S2). This finding aligns with the observations by Grekov et al. (60), where after 45 serial passages (24 days) in a nutrient-rich LB medium, PA accumulated a large number of mutations in genes related to the aforementioned functional categories. Notably, despite the absence of selection pressure, the evolved populations exhibited significantly increased cell densities during the stationary phase (60). This is also consistent with what we observed in the UmMd-evolved strain (60). Our evolution protocol also involved what Grekov et al. described as “weak bottlenecking,” which facilitates natural selection and increases the diversity and likelihood of fitness-enhancing evolutionary outcomes (60). As an opportunistic pathogen that must adapt to new habitats quickly, PA possesses high adaptation potential with a large genome for a bacterial species (5–7 Mbp) and consequently high phenotypic plasticity. Under the UmMd evolution conditions, PA was apparently able to explore the space of potential mutations to gain fitness, which explains the large number of observed mutations.

Phenotypically, the UmMd-evolved strain showed changes that were nearly opposite to those seen in SASn- and KPSn-evolved strains. For instance, compared to the ancestral strain, biofilm formation (Fig. 1b), pyocyanin production (Fig. 1c), relative fitness (Fig. 5b and c), and cell toxicity (Fig. 6a) all decreased in the UmMd-evolved strain. Simultaneously, the UmMd-evolved strain shows significantly fewer differential protein expressions compared to the SASn- and KPSn-evolved strains (Fig. 2a through c). The observed motility decline likely reflects adaptation to nutrient-rich conditions rather than NaCl as LB already contains NaCl, and our results are also consistent with prior reports of motility loss in LB-evolved PA (60). Altogether, this implies that the UmMd-evolved strain and SASn- and KPSn-evolved strains followed different evolutionary strategies and trajectories.

Prior works by us and others have explored the question of whether such evolution experiments are reproducible (61–63). Our previous work on *S. aureus* has shown that replicate bacterial populations did not display identical evolutionary trajectories due to inherent stochasticity in mutation and genetic drift (61). Therefore, although it is likely that replicate adaptive laboratory evolution experiments will produce different mutations, the phenotypic outcome of the evolution should be similar due to the selective pressure. The evolved strains should better adapt to the new environment, namely, the presence of the secretomes of the other species in our case. By studying the evolved strains, therefore, we should be able to gain insights into the adaptations of PA in the presence of SA and KP.

By comparing the proteomic profiles, we have identified many similarities with a few differences from the three evolved strains. The proteins related to O-antigen biosynthesis show significant upregulation only in the Sn-evolved strain (Fig. 3a through c). In a study by Tognon et al., after prolonged interactions with SA, *P. aeruginosa* strain PA14 generated mutations in the *orfN* gene (similar to glycosyltransferase WbpL of PAO1, which can affect both the synthesis of A- and B-bands), and caused the impairment of A-band and B-band lipopolysaccharide synthesis (30). However, our research indicated a distinct regulatory pattern where the downregulation of WbpM (specifically targeting B-band synthesis [51, 52]) and the upregulation of proteins (Rmd, Wzt, wbpY, specifically targeting A-band synthesis [52]) were exclusively observed in the SASn-evolved strain. Besides, Bittner et al. discovered in *Salmonella enterica* that RpoS and RpoN can regulate the production of O-antigen (64). In the SASn-evolved strain, a single-point mutation in *rpoS* resulted in the amino acid alteration (serine to isoleucine), which may be associated with the upregulation of proteins involved in O-antigen synthesis. These previous research and our study underscore the regulatory role of O-antigen biosynthesis changes in long-term bacterial interactions. Furthermore, the variant regulations of O-antigen may be influenced by different strains and evolutionary methods, and further investigation is needed to determine if these regulations associated with A-and B-band synthesis are specific to PA in the presence of SA.

Proteins associated with the T6SS, relevant to bacterial competitiveness, were differentially regulated in all evolved strains (Fig. 3a through c). Interestingly, the proteins related to both H2- and H3-T6SS were downregulated in the UmMd-evolved strain. In contrast, in the SASn- and KPSn-evolved strains, proteins related to H2-T6SS were upregulated, while those linked to H3-T6SS were downregulated. Moreover, the MvfR protein was upregulated in all three evolved strains. The MvfR protein, which typically affects H1-T6SS negatively and H2- and H3-T6SS positively (57), showed a different regulatory pattern in this case. This altered regulation could be a combined result of multiple gene mutations and a unique outcome of supernatant evolution. Besides, this regulation enhances the competitiveness of SASn- and KPSn-evolved strains during co-culture with SA and KP (57). This could imply an atypical regulation of the H2- and H3-T6SS clusters for PA in bacterial competition, especially in situations involving exposure to supernatants or secretions from other bacteria. Current research suggests that the T6SS not only impacts bacteria but may also affect eukaryotic cells (65, 66). Therefore, this regulation may be partially related to the higher cytotoxicity of SASn- and KPSn-evolved strains compared to UmMd-evolved and ancestral strains.

In summary, our research findings reveal the potential evolutionary adaptations of PA in prolonged coexistence with SA or KP. Through WGS and proteomic analysis, we uncovered the proteomic alterations due to genetic mutations in the evolved strains as well as key pathways involved in the adaptation of supernatant evolution. Furthermore, the atypical regulation of different clusters within the HSIs of T6SS present in both SASn- and KPSn-evolved strains and the distinct regulation of A- and B-bands of the O-antigen in SASn-evolved strains offer new insights into the long-term coculture of bacteria. These findings may provide actionable points for the prevention and treatment of polymicrobial infections.

## MATERIALS AND METHODS

### Bacterial strains and adaptive laboratory evolution in supernatant-conditioned media

In this study, bacterial strains utilized were *S. aureus* (MRSA) ATCC 43300, *K. pneumoniae* NRRL-B-3521, and *P. aeruginosa* PAO1.

We exposed PA to the cell-free supernatants of SA and KP, with modifications based on previous research (31, 67). Specifically, *P. aeruginosa* PAO1 was cultured in a 70:30 mixture of Luria Bertani broth (Sigma-Aldrich, Massachusetts, USA) and the sterile supernatants of either SA (SASn) or KP (KPSn) at the stationary phase, filtered through a 0.2 µm filter (Whatman, Buckinghamshire, UK). The control group received a 0.9% sodium chloride solution instead of the cell-free supernatant.

On the first day, *P. aeruginosa* was diluted to OD_600_ = 1.0 after overnight cultivation at 37°C with shaking at 220 rpm. This diluted culture was added at a 1:1,000 ratio to round-bottom polystyrene test tubes (Glendale, AZ, USA) containing 3 mL of the fresh medium with either 30% supernatants or NaCl solution. Each group had four replicate samples. Subsequently, the tubes were incubated at 37°C with shaking at 220 rpm. The culture underwent serial passaging for 15 cycles, each lasting 48 hours. At the conclusion of each cycle, a portion of the culture was re-inoculated into the fresh medium at a 1:1,000 ratio (LB with freshly prepared cell-free supernatant or 0.9% NaCl solution). The evolution experiment was stopped when visible phenotypic differences were detected. The single colonies of the strains were picked for the following experiments.

### Motility assay

We studied the twitching (68), swimming (69), and swarming (70) capabilities of the evolved *P. aeruginosa* PAO1. For twitching motility assessment, a solid medium was prepared with 25 g/L LB broth and 1% (wt/vol) Bacto agar (BD, New Jersey, USA) using subsurface stab assays. After overnight incubation at 37°C, the samples were subjected to crystal violet staining. Swimming motility was evaluated on a medium comprising 25 g/L LB and 0.3% (wt/vol) Bacto agar with point inoculation, and the plates were incubated at 37°C for 18 hours. To investigate swarming motility, a medium was created by mixing 8 g/L nutrient broth (Oxoid, Hampshire, UK), 5 g/L D-(+)-glucose (Sigma-Aldrich, Burlington, MA, USA), and 0.5% (wt/vol) Bacto agar, where a small volume of culture was spotted, and the plates were incubated at 37°C for 18 hours. The motility assay consisted of four replicate samples, and the experiment was repeated at least three times.

### Biofilm formation assay

The diluted overnight culture was added to a round-bottom 96-well dish containing the LB broth medium and incubated at 37°C for 48 hours. The liquid was removed by inverting the plate and immersing it in water to eliminate unattached cells and media components. A 0.1% crystal violet solution was added to each well and incubated for 15 minutes. The plate was gently washed with water and dried by inverting. To stain the biofilm, 30% acetic acid was added to dissolve the crystal violet. Then, the solution was transferred to new flat-bottom plates, and the absorbance at 550 nm was measured using a plate reader, and the data were normalized by bacterial CFUs.

### Pyocyanin production assay

For the extraction of pyocyanin, 1 mL of chloroform was added to 2 mL of the supernatant obtained from each PA strain. After vigorous vortexing for 2 minutes, resulting in the chloroform turning blue-green, the tubes underwent centrifugation at 3,000 × *g* at 4°C for 10 minutes. Subsequently, 300 µL of 0.2M hydrochloric acid was added to the partially extracted blue pyocyanin and vortexed vigorously for 2 minutes. The upper blue layer was acidified to a pink color, followed by an additional 2 minute centrifugation at 3,000 × *g* and 4°C. The absorbance of the fully extracted pyocyanin at the pink stage was measured at 520 nm and normalized by bacterial CFUs.

### Genomic DNA extraction, whole-genome sequencing, and data analysis

The bacterial populations at the end of the 15th cycle for all three evolution experiments, as well as randomly selected colonies, one from each population, were sent for whole-genome sequencing. The ancestral (PAO1) strain was also sequenced for verification of its genotype. Briefly, the extraction of genomic DNA from the evolved populations, as well as the overnight-cultured ancestral (PAO1) and the isolated strains (SASn-evolved, KPSn-evolved, and UmMd-evolved), was carried out utilizing the DNeasy blood and tissue kit (Qiagen) in accordance with the manufacturer’s guidelines. Subsequently, DNA was visualized via agarose gel electrophoresis and quantified employing a NanoVue Plus spectrophotometer (GE Healthcare).

For whole-genome sequencing library preparation, the quality of samples was meticulously checked based on the sample type and specific product requirements. Genomic DNA underwent fragmentation, end-repair, and 3′ adenylation, followed by ligation of adapters to the 3′ adenylated fragments. Subsequently, PCR was employed for product amplification, with the library quality control protocol tailored to product specifications. Single-stranded PCR products were denatured, circularized, and amplified through rolling cycle amplification to generate DNA nanoballs (DNBs) containing multiple DNA copies. High-quality DNBs were then loaded onto patterned nanoarrays using a DNBSEQ G400 instrument and sequenced in paired-end 150 bp format via combinatorial Probe-Anchor Synthesis (cPAS). After sequencing, raw reads underwent stringent filtering to eliminate adapter sequences, contamination, and low-quality reads. Quality control procedures were then implemented to assess the data integrity after the filtering process, ensuring the removal of any raw data with adapter sequences or low-quality sequences. This quality assessment stage involved a series of data processing steps carried out by the SOAPnuke software from BGI, with specific filtering parameters set to refine the data set and obtain valid, high-quality reads (71).

In the sequencing data analysis process, Snippy (version 4.6.0) was utilized to compare NGS sequence reads with a haploid reference genome, specifically using the complete genome of *P. aeruginosa* PAO1 (NCBI Reference Sequence: NC_002516.2), enabling the identification of substitutions (SNPs) and insertions/deletions (indels).

The formula was used to calculate mutation frequency in the evolved populations:


$$VAF\;=\;\frac{AO}{DP}$$

where VAF is the variant allele frequency, AO is the alternate allele observation count, and DP is the total read depth at the locus.

### Sample preparation for proteomics

After the supernatant evolution experiment, proteins from the exponential-phase cells of the ancestral strain, UmMd-evolved strain, SASn-evolved strain, and KPSn-evolved strain of *P. aeruginosa* were extracted. The ancestral strain was cultivated in LB broth, while the other three strains were cultured in the respective evolution experiment media at 37°C with shaking at 220 rpm overnight. The bacterial cells were prepared for protein sample extraction through three washes with PBS buffers.

Similar to our previous work (39), the cell pellet was resuspended in 300  µL of lysis buffer containing 8 M Urea and 50  mM Tris-HCl at pH 8.0. Subsequently, it was rapidly frozen in liquid nitrogen and subjected to sonication for 10 minutes. Following sonication, the sample underwent centrifugation at 16,000 × *g* for 10 minutes to eliminate cellular debris and insoluble components. A portion of the sample was set aside for BCA protein quantification assay (Pierce BCA Protein Assay Kit). The sample was treated with dithiothreitol (DTT) to a final concentration of 0.1 M and maintained at 37℃ for 1 h. In preparation for shotgun proteomics, 150  µg of proteins was combined with a maximum of 250  µL of exchange buffer containing 6 M urea, 50  mM Tris-HCl at pH 8.0, and 600 mM guanidine HCl. This mixture was then transferred to an Amicon filter device (Millipore, Darmstadt, Germany), and centrifuged at 14,000 × *g* for 20 minutes. The proteins within the filter device were treated with iodoacetamide (IAA) at a concentration of 50  mM in the exchange buffer for 20 minutes in darkness, followed by centrifugation at 14,000 × *g* for another 20 minutes. To reduce the urea concentration, 250  µL of 50  mM ammonium bicarbonate was added to the filter device and centrifuged at 14,000 × *g* for 20 minutes. This step was repeated once. Protein digestion was carried out using sequencing-grade modified trypsin at a ratio of 1:50 (wt/wt) (Promega, Madison, WI), for 12 hours at 37°C. Subsequently, the sample was acidified with 10% formic acid to achieve a final concentration of 0.1% (vol/vol) and centrifuged at 16,000 × *g* for 5 minutes. Finally, the samples underwent desalting using a C18 reverse-phase ZipTip (Millipore in Darmstadt, Germany) and were dried using a SpeedVac (Eppendorf, Hamburg, Germany) for 30 minutes.

### Liquid chromatography

The protein samples were dissolved in a blend of water-acetonitrile-formic acid at a ratio of 97.9:2:0.1 and processed through a Bruker nanoElute ultrahigh-performance liquid chromatograph (UHPLC) linked to a TimsTOF Pro hybrid trapped-ion mobility quadrupole time-of-flight mass spectrometer (Bruker Daltonics, Bremen, Germany), utilizing a nanoelectrospray ion source known as CaptiveSpray. A sample volume of 1 µL (approximately containing 200 ng of protein digest) was injected into the UHPLC system and separated on an IonOpticks 25 cm Aurora Series Emitter column with a Captive Spray insert (250 mm by 75 µm internal diameter, 120 Å pore size, and 1.6 µm particle size C18) at a flow rate of 0.3 mL/min. The mobile phase included 0.1% formic acid in water for solvent A and 0.1% formic acid in acetonitrile for solvent B. The gradient transitioned from 2% to 5% solvent B within 0.5 min, from 5% to 30% solvent B over 26.5 min, and finally from 30% to 95% solvent B within 0.5 min. Following a 0.5 min retention at 95% solvent B, the mobile phase was reduced to 2% solvent B in 0.1 min. A 2% solvent B equilibration phase occurred for 2 min before the next injection.

### TimsTOF pro mass spectrometer

In a concise overview, upon entering the first vacuum stage, ions from the CaptiveSpray ion source undergo a 90° deflection before being stored within the foremost segment of a dual trapped-ion mobility spectrometry (TIMS) analyzer. A radio frequency (RF) potential of 300 Vpp is utilized for radial trapping of the ion cloud. After the initial phase of accumulation, the ions are transferred to the secondary segment of the TIMS analyzer to undergo parallel assessment of ion mobility. In both areas of the TIMS analyzer, an increasing longitudinal electric field gradient is superimposed onto the RF voltage, propelling the ions within the channel with the incoming gas flow while repelling them with the electric field simultaneously. As the electric field gradient decreases, ions are sequentially liberated from the TIMS analyzer according to their individual ion mobilities, facilitating subsequent quadrupole time-of-flight (QTOF) mass analysis. The dual TIMS setup permits operation at a 100% duty cycle as long as the accumulation and ramp times are maintained in equilibrium. In this study, we configured the accumulation and ramp durations at 100 ms each. Mass spectra were obtained in the positive electrospray mode over the *m/z* range of 100 to 1,700. Ion mobility was examined between 0.85 and 1.30 V*s/cm^2^. The quadrupole isolation width was set at 2 Th for *m/z* values below 700 and 3 Th for those exceeding 700, while the collision energy increased linearly from 27 eV to 45 eV in accordance with the ascending ion mobility. The entire acquisition cycle, lasting 0.53 s, included a full TIMS-MS scan and four subsequent accumulation-serial fragmentation (PASEF) tandem mass spectrometry (MS/MS) scans. Low-abundance precursor ions, exceeding 2,500 counts but below a target intensity of 20,000 counts, were repeatedly scheduled and dynamically excluded for 0.4 minutes. Calibration of the TIMS dimension was linearly performed using three specific ions from the Agilent electrospray ionization (ESI) LC-MS tuning mix (*m/z*, 1/K0: [622.0289, 0.9848 Vs*cm^−2^], [922.0097, 1.1895 Vs*cm^−2^], and [1221.9906, 1.3820 Vs*cm^−2^]) in the positive mode. An analysis with details of the mass spectrometer can be accessed in prior literature (72, 73).

### Database searching and label-free quantification of proteomics data

The initial step involved converting the raw data obtained from the TimsTOF Pro mass spectrometer into mgf files, which were then transformed into mzXML files using ProteoWizard’s msconvert program (version 3.0.11676 64-bit) (74). Subsequently, the mzXML files underwent analysis through the Comet open-source tool (version 2019.04 rev.0) (75) within the Trans-proteomics pipeline (version 5.1.0) (75), against the *P. aeruginosa* PAO1 protein sequence database sourced from UniProt (SWISSprot). Decoy sequences were generated by scrambling amino acid sequences amid tryptic cleavage sites and amalgamated into the database. These decoy sequences were employed to evaluate the peptides identified, regulated by false discovery rate (FDR). The Comet search parameters were configured with specifics including a 40 ppm peptide mass tolerance, monoisotopic mass type, fully digested enzyme termini, 0.05 amu fragment bin tolerance, 0 amu fragment bin offset, oxidized methionine, and carbamidomethylated cysteine as the variable and fixed modifications. Post-Comet analysis, the results underwent further processing via PeptideProphet (76), iProphet, and ProteinProphet integrated into the Trans-Proteomics Pipeline (TPP) (75) employing the decoy-assisted nonparametric mode. Each mzXML run was individually scrutinized, with protein identifications being sieved at an FDR threshold of 0.01.

In this study, the quantification methodology employed was based on NSAF (77):


$${\left(NSAF\right)}_{k}=\frac{SpC_{k}/L_{k}}{\sum_{i=1}^{N}\left(SpC_{i}/L_{i}\right)}$$

where ${SpC}_{k}$ is the total tandem MS spectra matched to protein *k*, $L_{k}$ ia the amino acid sequence length of protein *k*, and *N* is the number of proteins quantified. Only proteins identified across a minimum of two out of three biological replicates were quantified. Differentially expressed proteins were sieved using specific criteria: an average spectral count of at least three, *P*-values derived from Student’s *t*-test on NSAF values below 0.05, and fold changes exceeding or falling below 1.5-fold. Here, the fold changes of the evolved strains are defined as the NSAF of evolved strains divided by the NSAF of the ancestral strain.

### Bioinformatics analysis for proteomics

Each evolved strain was compared with the ancestral strain to reveal their post-evolution characteristics. Pairwise comparisons were then conducted among the evolved strains to examine the similarities and differences in protein expression under different growth environments.

The logarithm-transformed NSAF values were employed in Python using the PCA function from the sklearn package with centering and scaling. Predicted protein-protein interaction (PPI) data were obtained from STRING (version 11.0; highest confidence). For gene annotation and enrichment analysis, Database for Annotation, Visualization and Integrated Discovery (DAVID, version 6.8) was utilized for pathway and functions analysis. The results of the enrichment analysis were visualized using the R program (version 4.4.0). The log_2_ fold change (evolved strains vs ancestral strain) for each protein was utilized to generate the heatmap. The visualization, including the heatmap and the clustering of rows and columns, was conducted using the pheatmap package in R. Cytoscape software (version 3.8.1) was utilized for visualizing all networks. Gene ontology (GO), Kyoto Encyclopedia of Genes and Genomes (KEGG), and InterPro were used as the databases.

### Disk diffusion antimicrobial susceptibility test

The disk diffusion test followed the standard EUCAST susceptibility test guideline (78), involving the application of an overnight cultured inoculum suspension equivalent to a 0.5 McFarland standard (approximately 1–2 × 10^8^ CFU/mL) spread on MH agar. This was then treated with antimicrobial disks containing carbomycin (100 µg), kanamycin (60 µg), tetracycline (30 µg), and ampicillin (100 µg) and incubated at 35°C for 20 h.

### Relative fitness

The ancestral strains and evolved strains of PA, along with SA and KP, were overnight-cultured and adjusted to an OD_600_ of 1.0. Following a 1,000-fold dilution in LB broth, they were mixed in a ratio of 1:100 for PA to SA or KP. The mixed cultures were then incubated at 37°C with shaking at 220 rpm for 24 hours. The bacterial CFUs were calculated before and after coculturing. The relative fitness of PA was determined using two formulas (79):

Relative fitness of PA to SA = $ln\;\left[\left({PA}_{end}/{SA}_{end}\right)/\left({PA}_{start}/{SA}_{start}\right)\right]$

Relative fitness of PA to KP = $ln\;\left[\left({PA}_{end}/{KP}_{end}\right)/\left({PA}_{start}/{KP}_{start}\right)\right]$

A relative fitness value greater or less than 0 indicates either success or failure in competition against SA or KP.

### Growth curve of PA strains

The ancestral strains and evolved strains of PA were cultured overnight and adjusted to an OD_600_ of 1.0. After a 1,000-fold dilution in LB broth, 200 µL of the bacterial suspension was added to a 96-well plate, with four replicates for each strain. The plate was then incubated at 37°C with shaking at 220 rpm for 30 h. OD_600_ readings were taken at 0, 2, 4, 5, 6, 7, 8, 9, 10, 11, 12, 13, and 30 h. Prior to fitting the growth curve, OD_600_ values from the cell-free medium were subtracted from all measurements. The minpack.lm package in R was used for fitting the growth curve. The fitting was based on the following logistic growth model:


$$N\left(t\right)=\frac{K}{1+\left(\frac{K-N_{0}}{N_{0}}\right)e^{-rt}}$$

where *N*(*t*) is the population density at time *t*, *K* is the maximum carrying capacity, *r* is the growth rate constant, and *N*_0_ is the initial population density.

### Cytotoxicity

HeLa cells were used for studying bacterial cytotoxicity. The HeLa cells were cultured in high-glucose DMEM containing 10% FBS. The overnight-cultured ancestral strains and evolved strains of PA were diluted in fresh cell culture medium, and 2 mL/well bacterial suspension was added to the HeLa cells in a six-well plate at an MOI of 100 after prior washing of the cells with PBS. The cell-bacteria co-culture plate was centrifuged at 1,000 × *g* for 5 minutes and then incubated at 37°C, 5% CO_2_, and 99% humidity. After a 10 h infection period, 300 µL of the cell supernatant was transferred and kept in a separate tube. The cells and bacterial debris were removed by centrifugation. An LDH Cytotoxicity Assay Kit (Thermo Fisher, Massachusetts, USA) was used to measure LDH levels following the instructions provided. The remaining supernatant and cells were thoroughly mixed using a pipette to calculate the bacterial CFU per well, thus eliminating the influence of the maximum carrying capacity of bacterial populations on cytotoxicity.

## Data Availability

The mass spectrometry proteomics data have been deposited to ProteomeXchange through PRIDE with the data set identifier PXD059669. Whole-genome sequence data of ancestral, UmMd-evolved, SASn-evolved, and KPSn-evolved strains have been deposited in the BioProject database under the accession number PRJNA1210149.

## SUPPLEMENTAL MATERIAL

Supplemental material msystems.00111-25-s0001.docx Fig. S1 to S6 and Tables S1 and S2.

ASM does not own the copyrights to Supplemental Material that may be linked to, or accessed through, an article. The authors have granted ASM a non-exclusive, world-wide license to publish the Supplemental Material files. Please contact the corresponding author directly for reuse.
