# Supplementary material for: Proteomic study of evolved Pseudomonas aeruginosa strains grown in Staphylococcus aureus- and Klebsiella pneumoniae-conditioned media
Source: mSystems. 2025 Jun 3;10(7):e00111-25. doi: 10.1128/msystems.00111-25 (PMC12282091; doi:10.1128/msystems.00111-25)
Supplement: Supplemental material — Fig. S1 to S6 and Tables S1 and S2. [file msystems.00111-25-s0001.docx]

**Supplemental Material**


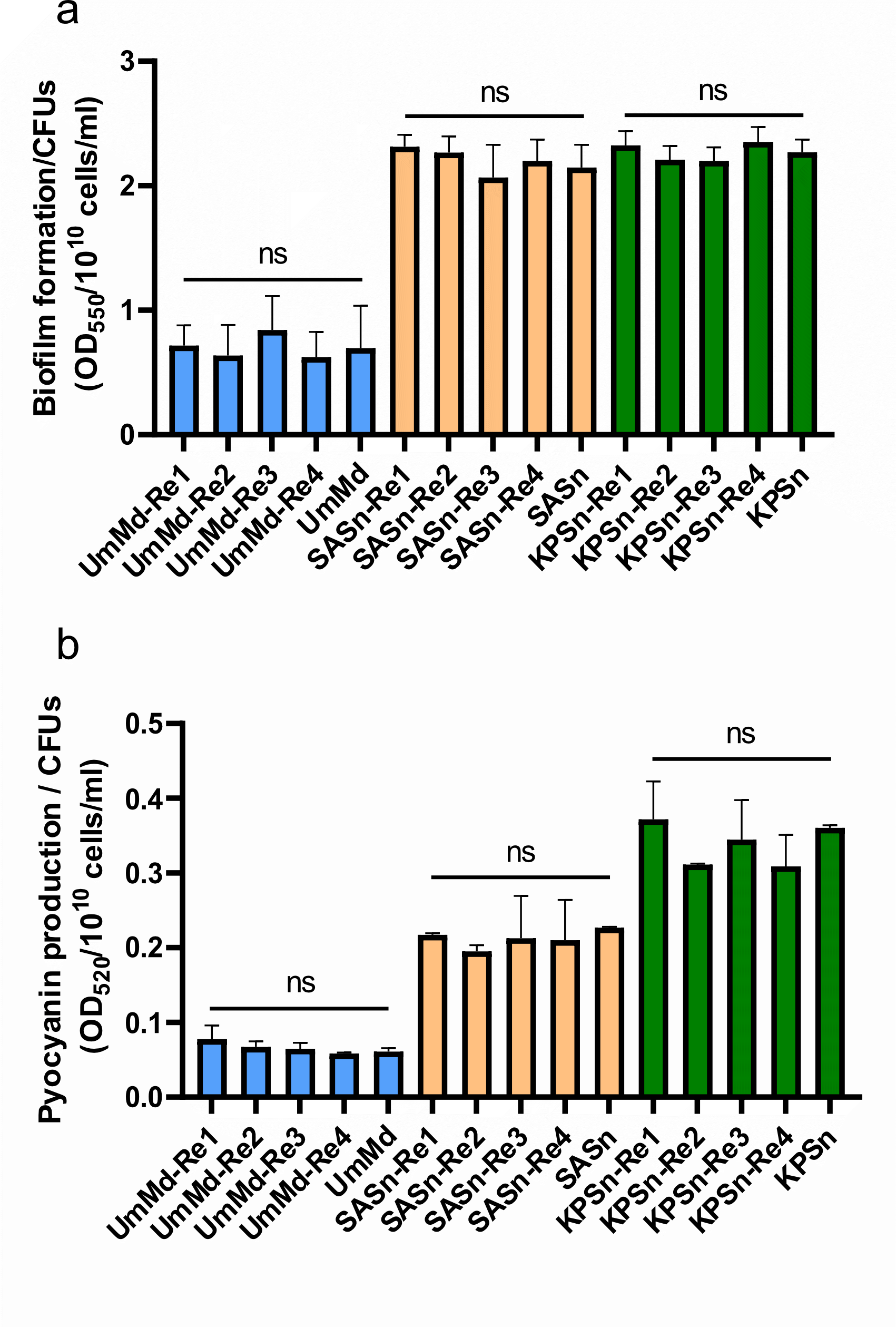


**Figure S1.** Phenotypes of evolved PA strains cultured in fresh LB medium. The 3 evolved strains (UmMd-, SASn- and KPSn-evolved) were further cultured with four biological replicates (Re1-4) in LB medium for 14 days (7 growth cycles). The biofilm formation (a) and pyocyanin production (b) of re-cultured strains were compared with the pre-cultured ones (mean ± s.d., n = 4). Significance of difference: ns, not significant, ∗*p* < 0.05, ∗∗*p* < 0.01, ∗∗∗*p* < 0.001 (one-way ANOVA with correction for multiple testing via the Brown–Forsythe test).


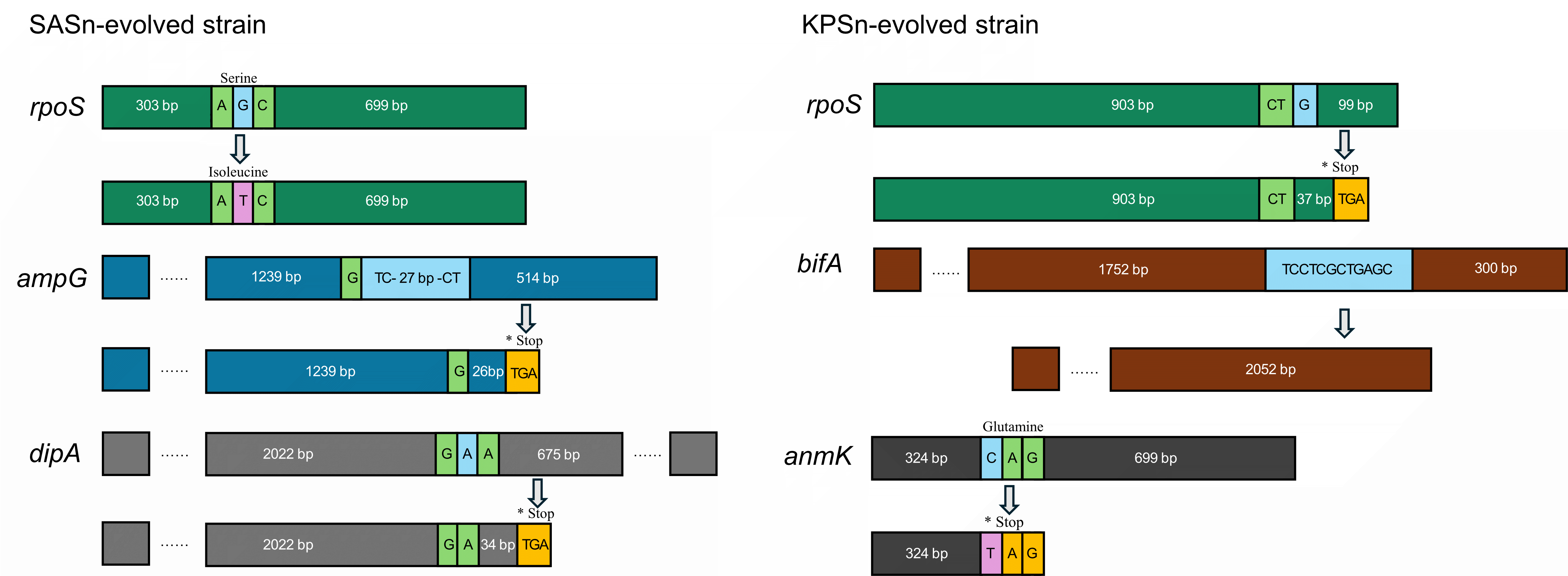


**Figure S2**. Non-synonymous mutations identified in Sn-evolved strains of PA. Cyan-highlighted regions denote altered or deleted nucleotide sequences, with amber-colored zones marking the emergence of premature stop codons.

**Table S1.** Non-synonymous mutations identified in the KPSn-evolved PA population at above 15% VAF, but not in the isolated strain. Asterisks (*) indicate nucleotide deletion that results in a frame shift and a premature stop codon downstream of the deletion.

| **Position** | **REF** | **ALT** | **Protein change** | **Gene** | **VAF (%) of population** | **VAF (%) of isolated KPSn strain** |
| --- | --- | --- | --- | --- | --- | --- |
| 4057959 | G | A | Arginine (R) → Tryptophan (W) | *rpoS* | 33.8 | 0 |
| 5643032 | GA | G | Loss of 213 amino acids* | *dipA* | 37.1 | 0 |

**Table S2**. Non-synonymous mutations identified in UmMd-evolved population and isolated strain, at above 15% VAF in the population

| **Strain** | **Position** | **REF** | **ALT** | **Protein change** | **Gene** | **Function** | **VAF (%) of population** | **VAF (%) of isolate** |
| --- | --- | --- | --- | --- | --- | --- | --- | --- |
| UmMd | 1924149 | C | T | Proline (P) → Leucine (L) | *PA1779* | Assimilatory nitrate reductase | 71.5 | 100 |
|  | 1979098 | C | T | Histidine (H) → Tyrosine (Y) | *nhaB* | Na(+)/H(+) antiporter NhaB | 65.2 | 100 |
|  | 2080884 | C | T | Alanine (A) → Valine (V) | *PA1909* | Hypothetical protein | 62.5 | 100 |
|  | 2141484 | A | G | Serine (S) → Alanine (A) | *PA1956* | Hypothetical protein | 57.4 | 100 |
|  | 2438101 | A | G | Glutamic acid (E) → Glycine (G) | *PA2217* | Aldehyde dehydrogenase | 74.1 | 100 |
|  | 3026536 | G | A | Arginine (R) → Glutamine (Q) | *PA2679* | Hypothetical protein | 64.1 | 100 |
|  | 3190144 | T | C | Valine (V) → Alanine (A) | *PA2836* | Secretion protein | 62.3 | 100 |
|  | 3239238 | C | T | Alanine (A) → Valine (V) | *atuA* | Hypothetical protein | 58.3 | 100 |
|  | 3440480 | G | A | Alanine (A) → Threonine (T) | *gdhB* | NAD-specific glutamate dehydrogenase | 58.1 | 100 |
|  | 3761499 | G | A | Alanine (A) → Threonine (T) | *PA3349* | Chemotaxis protein | 93.1 | 100 |
|  | 4111289 | G | A | Arginine (R) → Glutamine (Q) | *PA3672* | ABC transporter APT-binding protein | 63.2 | 100 |
|  | 5146372 | T | C | Methionine (M) → Threonine (T) | *PA4594* | ABC transporter APT-binding protein | 68.4 | 100 |
|  | 5230587 | T | C | Tryptophan (W) → Arginine (R) | *murI* | Glutamate racemase | 54.4 | 100 |
|  | 5551483 | T | TGCCGCGACC | Insertion of 3 amino acids | *mutL* | DNA mismatch repair protein | 83.5 | 100 |
|  | 5814564 | GC | G | Frame shift | *PA5165* | Two-component sensor | 54.9 | 100 |
|  | 6151035 | G | A | Alanine (A) → Threonine (T) | *PA5459* | Hypothetical protein | 64.8 | 100 |
|  | 1355220 | T | C | **Leucine (L) → Proline (P)** | *aprF* | Alkaline protease secretion outer membrane protein AprF precursor | 56.8 | 0 |
|  | 740432 | A | G | **Histidine (H) → Arginine (R)** | *PA0683* | Type II secretion system protein | 57.9 | 0 |
|  | 2952043 | A | G | **Phenylalanine (F) → Leucine (L)** | *cysG* | Siroheme synthase | 57.9 | 0 |
|  | 4412101 | T | C | **Asparagine (N) → Isoleucine (I)** | *PA3934* | Conserved hypothetical protein | 58.1 | 0 |
|  | 5149395 | G | A | **Glycine (G) → Aspartic acid (D)** | *PA4596* | Transcription regulatory; DNA binding | 59.7 | 0 |
|  | 1690393 | C | T | **Glycine (G) → Serine (S)** | *PA1552* | Cytochrome c oxidase, cbb3-type, CcoP subunit | 60.6 | 0 |
|  | 4984590 | G | A | **Alanine (A) → Glutamic acid (E)** | *PA4450* | UDP-N-acetylglucosamine 1-carboxyvinyltransferase | 61.0 | 0 |
|  | 1542239 | C | T | **Alanine (A) → Threonine (T)** | *PA1417* | Probable decarboxylase | 61.0 | 0 |
|  | 1183386 | C | T | **Alanine (A) → Valine (V)** | *PA1092* | Flagellin type B | 61.6 | 0 |
|  | 5045965 | T | C | **Valine (V) → Alanine (A)** | *PA4505* | Dipeptide ABC transporter ATP-binding protein DppD | 61.7 | 0 |
|  | 1570635 | C | T | **Threonine (T) → Isoleucine (I)** | *PA1441* | Putative flagellar hook-length control protein FliK | 62.0 | 0 |
|  | 862042 | A | G | **Leucine (L) → Histidine (H)** | *PA0788* | Hypothetical protein | 68.0 | 0 |
|  | 3493955 | G | A | **Proline (P) → Serine (S)** | *PA3113* | N-(5'phosphoribosyl) anthranilate (PRA) isomerase | 70.5 | 0 |
|  | 1770693 | C | T | **Glycine (G) → Arginine (R)** | *PA1627* | Probable transcriptional regulator | 93.8 | 0 |


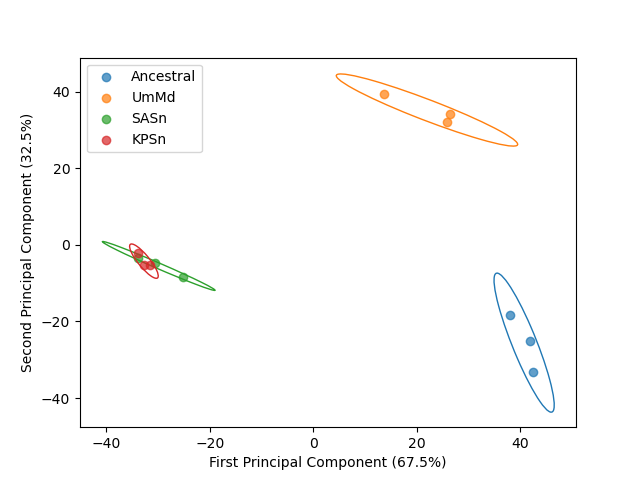


**Figure S3**. Principal component analysis (PCA) of the proteomes of the UmMd-, SASn-, KPSn-evolved strains and the ancestral strain, using NSAF as a quantitative measure of protein abundance.


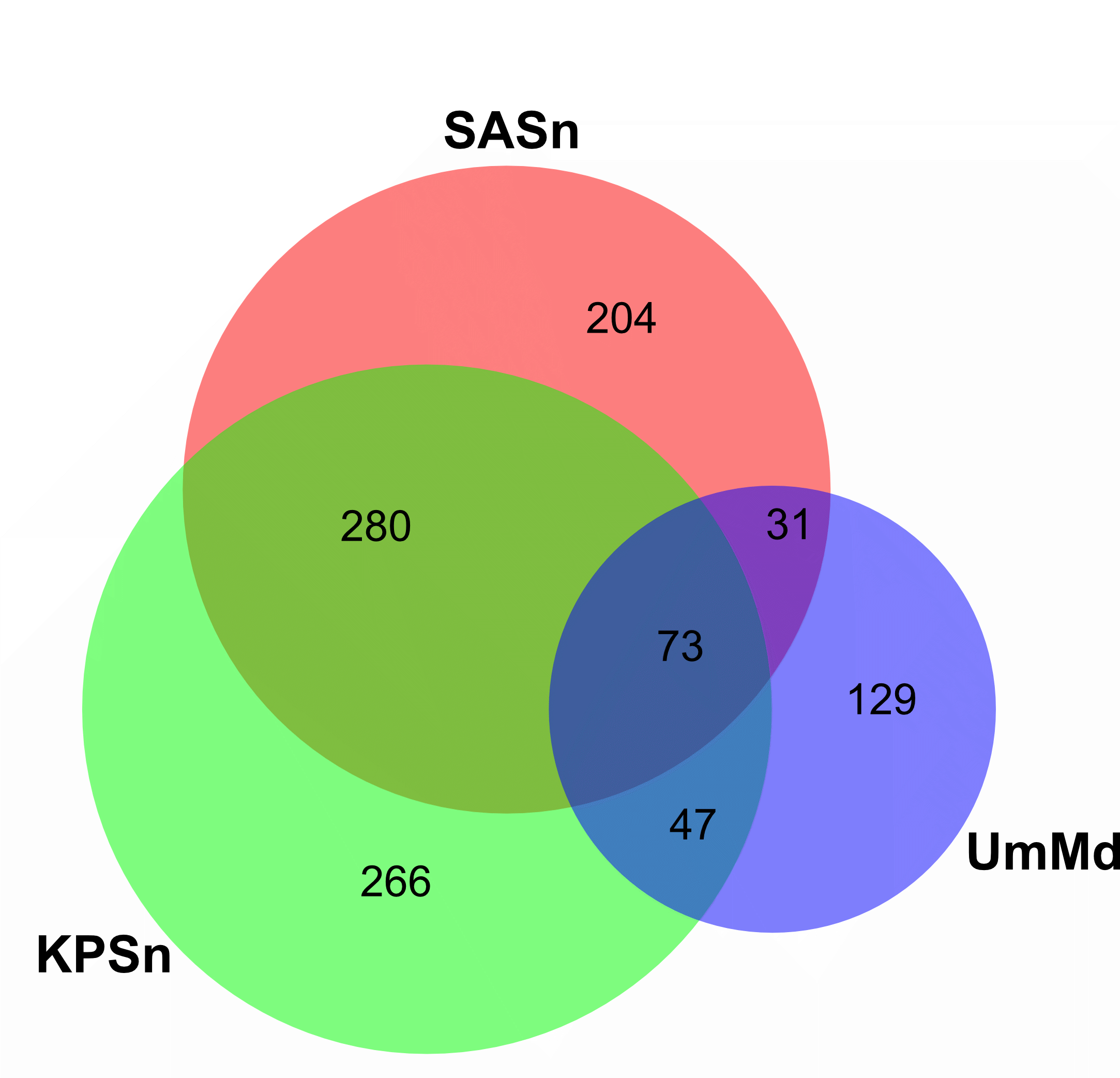


**Figure S4.** Venn diagram of DEPs. Red, green, and blue circles represent DEPs specific to the UmMd-evolved, SASn-evolved, and KPSn-evolved strains, respectively. Overlapping regions denote shared DEPs across strains.


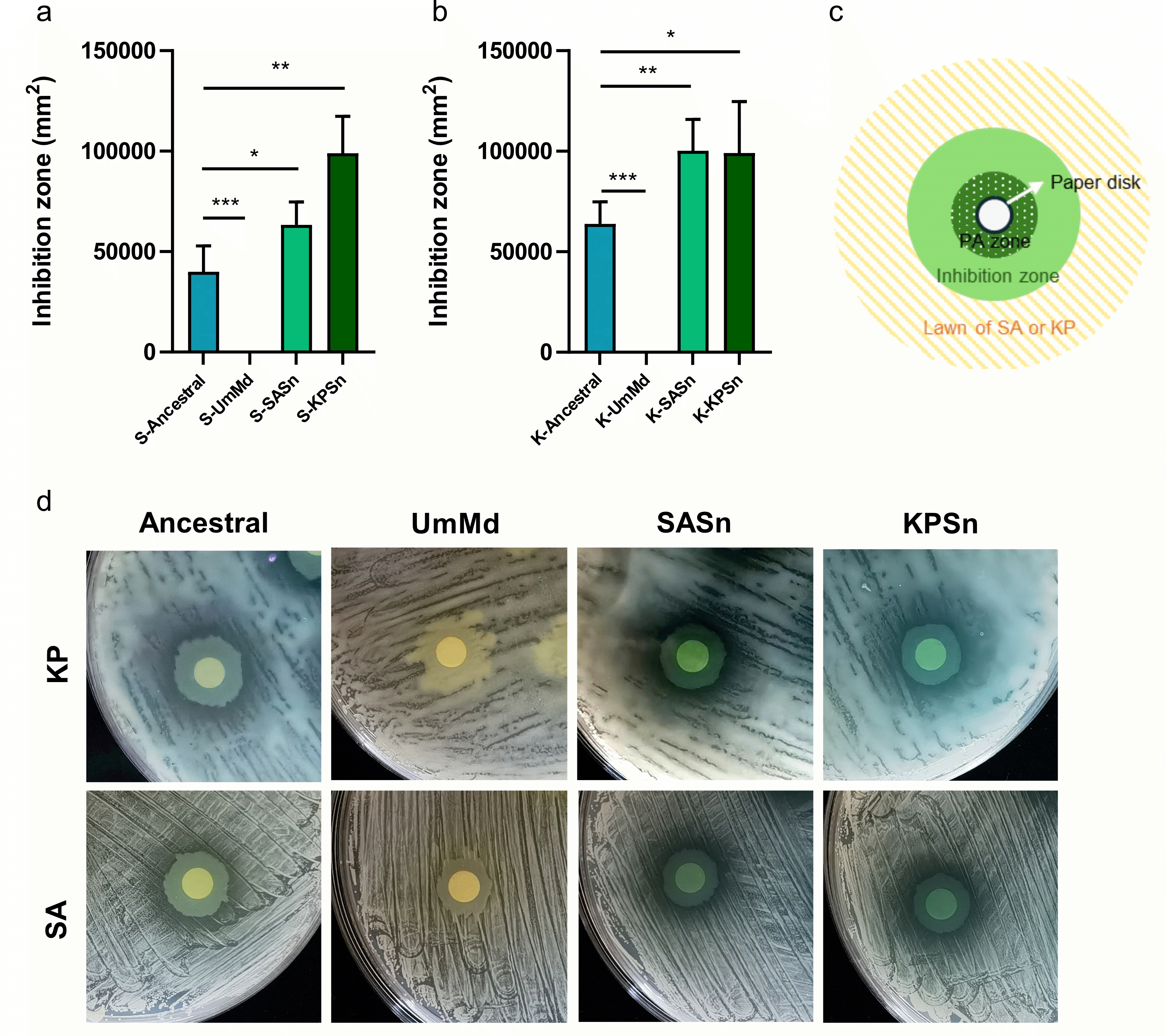


**Figure S5.** The zone of inhibition formed by evolved PA strains and ancestral strain on the lawn of SA or KP (a, b, d) The zone of inhibition, measured by ImageJ (mean ± s.d., n = 4). (c) Illustration of inhibition zone area calculation, with the light green area being the region of interest for measurement. Significance of difference: ns, not significant, ∗*p* < 0.05, ∗∗*p* < 0.01, ∗∗∗*p* < 0.001 (two-tailed *t*-test with unequal variances). The diameter of the paper disks used to carry PA at beginning were 6mm


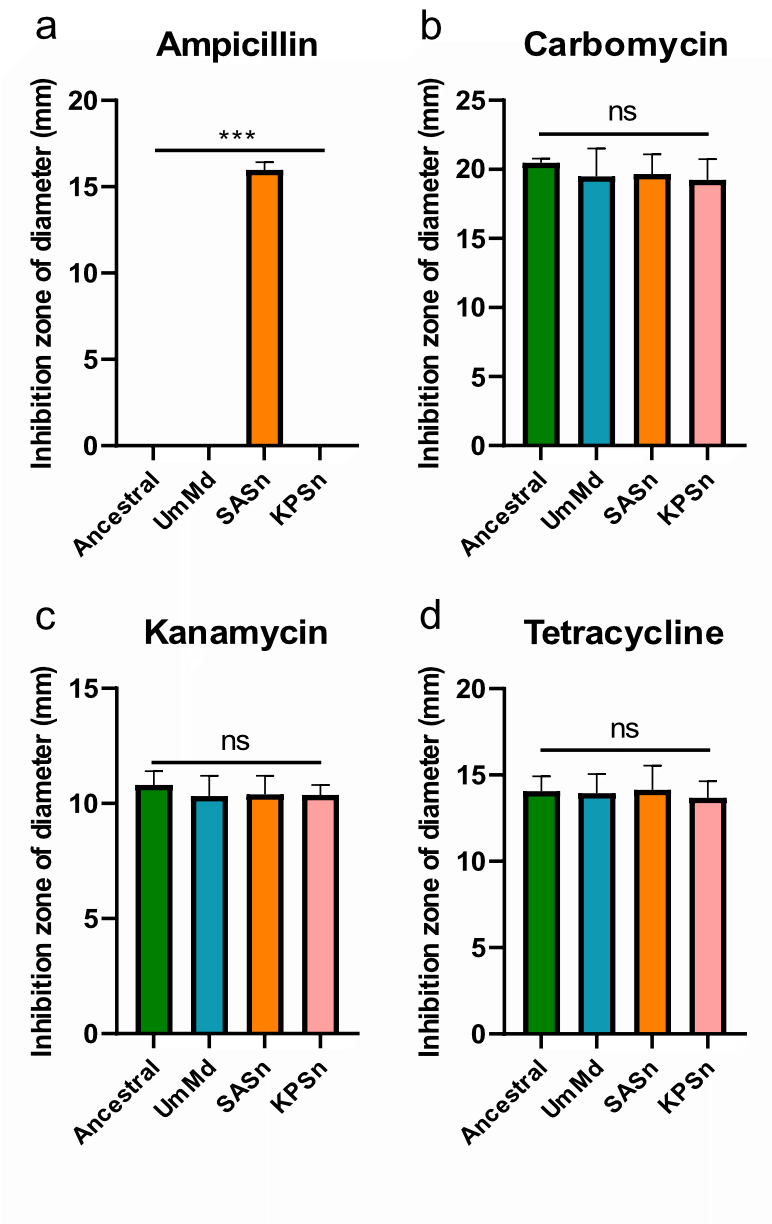


**Figure S6.** The inhibition zone of diameter formed by disk papers containing ampicillin, carbomycin, kanamycin, and tetracycline (a-d) on the lawn of ancestral strain and evolved strains. The zone of inhibition, measured by ImageJ (mean ± s.d., n = 4). Significance of difference: ns, not significant, ∗*p* < 0.05, ∗∗*p* < 0.01, ∗∗∗*p* < 0.001 (one-way ANOVA with correction for multiple testing via the Brown–Forsythe test)
